# Development and validation of a novel nomogram for prediction of ketosis-prone type 2 diabetes

**DOI:** 10.3389/fendo.2023.1235048

**Published:** 2023-09-27

**Authors:** Rui Min, Yiqin Liao, Bocheng Peng

**Affiliations:** ^1^ Department of Geriatrics, Wuhan Fourth Hospital, Wuhan, Hubei, China; ^2^ Department of Thyroid and Breast Surgery, Xianning Central Hospital, Xianning, Hubei, China; ^3^ Department of Pain, Wuhan Fourth Hospital, Wuhan, Hubei, China

**Keywords:** ketosis-prone type 2 diabetes, nomogram model, prediction, risk factors, glycosylated hemoglobin A1c, free fatty acid

## Abstract

**Background:**

Ketosis-prone type 2 diabetes (KPD), as a unique emerging clinical entity, often has no clear inducement or obvious clinical symptoms at the onset of the disease. Failure to determine ketosis in time may lead to more serious consequences and even death. Therefore, our study aimed to develop and validate a novel nomogram to predict KPD.

**Methods:**

In this retrospective study, clinical data of a total of 398 newly diagnosed type 2 diabetes in our hospital who met our research standards with an average age of 48.75 ± 13.86 years years old from January 2019 to December 2022 were collected. According to the occurrence of ketosis, there were divided into T2DM groups(228 cases)with an average age of 52.19 ± 12.97 years, of whom 69.74% were male and KPD groups (170cases)with an average age of 44.13 ± 13.72 years, of whom males account for 80.59%. Univariate and multivariate logistic regression analysis was performed to identify the independent influencing factors of KPD and then a novel prediction nomogram model was established based on these independent predictors visually by using R4.3. Verification and evaluation of predictive model performance comprised receiver-operating characteristic (ROC) curve, corrected calibration curve, and clinical decision curve (DCA).

**Results:**

4 primary independent predict factors of KPD were identified by univariate and multivariate logistic regression analysis and entered into the nomogram including age, family history, HbA1c and FFA. The model incorporating these 4 predict factors displayed good discrimination to predict KPD with the area under the ROC curve (AUC) of 0.945. The corrected calibration curve of the nomogram showed good fitting ability with an average absolute error =0.006 < 0.05, indicating a good accuracy. The decision analysis curve (DCA) demonstrated that when the risk threshold was between 5% and 99%, the nomogram model was more practical and accurate.

**Conclusion:**

In our novel prediction nomogram model, we found that age, family history, HbA1c and FFA were the independent predict factors of KPD. The proposed nomogram built by these 4 predictors was well developed and exhibited powerful predictive performance for KPD with high discrimination, good accuracy, and potential clinical applicability, which may be a useful tool for early screening and identification of high-risk population of KPD and therefore help clinicians in making customized treatment strategy.

## Introduction

As one of the serious acute medical hyperglycemic emergency of diabetes mellitus, ketosis has been largely considered as an unique initial clinical manifestation of type 1 diabetes to a large extent because of the serious and irreversible insulin deficiency due to disorders of the autoimmune system (1). However, In recent decades, there is increasing evidence suggests that ketosis can also occur in type 2 diabetes as well (2). An emerging subtype of diabetes, known as ketosis-prone type 2 diabetes (KPD), which shares a similar pathophysiology as type 2 diabetes and does not necessarily meet the typical features of autoimmune type 1 diabetes has been well defined (3). Patients with KPD are usually overweight or obese young and middle-aged males, have a high frequency of family history with type 2 diabetes, present with elevated plasma glucose levels of 500-700 mg/dl as well as positive blood and urine ketone bodies and different from type 1 diabetes, it do not exibit autoantibodies against islet beta cells (4). The main pathogenesis of KPD is β-cell dysfunction manifesting as a short-term insulin deficiency based on severe insulin resistance that causes severe hyperglycemic and ketosis (5). Unlike ketosis seen in type 1 diabetes, these subjects often have no clear inducement and obvious clinical symptoms initially, which may be the reason for the greatly underestimated prevalence (6). According to reports, after several weeks to months of active initial hypoglycemic treatment with insulin, many of the subjects with KPD become insulin independent and their abnormal blood glucose levels can be well controlled through lifestyle and dietary changes alone or with oral hypoglycemic medication (7). If the management is quite poor or the precipitating factors are not addressed, severe and persistent glucotoxicity will lead to dysfunction of β-cell function, which will form a vicious circle, resulting in a high mortality rate in patients with KPD (8).

Currently, the diagnosis of KPD relies heavily on the detection of blood or urine ketone bodies (9). While this method can indicate the presence of ketosis, it is unable to identify individuals who may be at a higher risk of developing the condition (10). Various studies, both domestic and foreign, have identified certain risk factors associated with KPD (11). However, there is a need for a quantitative method that can effectively evaluate the risk of KPD and enable more precise and efficient clinical predictions. The Nomogram model is capable of integrating multiple independent predict factors and presenting them in a visual manner. By calculating scores, it provides a more intuitive, clear, and quantitative prediction of the probability of disease occurrence, enabling effective risk assessment (12).

Therefore, this study aims to establish and verify a Nomogram model for predicting the risk of KPD, so as to screen and identify high-risk groups of KPD as early as possible in a convenient and intuitive way, and provide clinical reference for prevention and treatment of acute complications of diabetes.

## Methods and materials

### Study design and participants

We conducted this retrospective case analysis study based on data collected from our previous studies and data collected from open source databases (13). In this retrospective study, we strictly followed the inclusion and exclusion criteria and finally enrolled 398 patients with initial diagnosis of type 2 diabetes (18~88 years old, mean age: 48.75 ± 13.86 years), who were hospitalized in our hospital from January 2019 to December 2022. According to the definition and criteria for diabetes set by the America Diabetes Association (ADA) of 2019, type 2 diabetes was defined by fasting blood glucose (FBG)≥7.0 mmol/L and/or 2h-postprandial plasma glucose (2h PG) >11.1mmol/L during a 75 g oral glucose tolerance test. The inclusion criteria were as follows: (1) Newly diagnosed with untreated type 2 diabetes, the course of disease is within half a year; (2) KPD patients with ketosis were defined as positive blood ketones (β-hydroxybutyric acid > 0.30 mmol/L) and/or moderate to heavy urine ketones (urine ketones ≥ (2+)), and in the absence of glutamic acid decarboxylase antibody (GAD) and insulin autoantibody (IAA); T2DM without ketosis were defined as diabetes with neither diabetic ketosis (β-hydroxybutyric acid ≤ 0.30mmol/L and ketone body (-)) nor GAD or IAA autoantibodies. The exclusion criteria were as follows: (1) Patients with severe infection, acute myocardial infarction, acute cerebral infarction, kidney injury, delirium, seizures, acute alcohol intoxication, pregnancy diabetes, severe trauma, malignant tumor, surgery, corticoid therapy, etc., which might result in ketosis; (2) Patients with diabetic ketoacidosis; (3) Patients who have taken any hypoglycemic drugs or other drugs that may affect glucose and lipid metabolism; (4) Patients with severe liver function or renal insufficiency; (5) Secondary diabetes, such as pancreatic exocrine diseases and other endocrine diseases; (6) Patients with incomplete clinical data. A total of 398 patients with newly diagnosed T2DM were finally enrolled in this study, 50 patients with positive islet-related autoantibodies were excluded, and 83 patients who did not meet the other criteria of the study were also excluded. Of the 398 T2DM patients, 170 (42.71%) were diagnosed for KPD and 228 (57.29%) were diagnosed for non-ketotic T2DM. During the patient’s hospitalization, T2DM group were treated with insulin and/or oral hypoglycemic drugs, and for the KPD group, all patients received intensive insulin therapy, including external insulin infusion pump or insulin injection at least three times a day, and intravenous fluids, electrolytes etc.

The study complies with the Declaration of Helsinki and has been approved by the Ethics Committee of Wuhan Fourth Hospital (KY2023-067-01).

### Data collection

General clinical data of patients were collected through a comprehensive and careful consultation by professional doctors, including basic information such as age, sex, present and past medical history. Nurses with standardized training underwent a simple physical examination, including the measurement of height (m) and body weight (kg) of all patients, from which the body mass index (BMI) = kg/m²was measured, and overweight/obesity was defined as BMI≥25 kg/m^2^ for Asian population. When the patient first visited a doctor, the random blood glucose of the finger or vein, blood β-hydroxybutyric acid, blood lactic acid, urine routine, arterial blood gas analysis and other indexes were urgently checked. The inpatients were instructed to keep fasting for at least eight hours before exsanguinate assay in the next morning. The venous blood samples collected including GAD-Ab, IAA-Ab, fasting plasma glucose (FPG), glycosylated serum protein (GSP), glycosylated hemoglobin A1C (HbA1c), total triglycerides (TG), total cholesterol (TC), high-density lipoprotein cholesterol (HDL-C), low-density lipoprotein cholesterol (LDL-C), lipoprotein a [LP (a)], serum uric acid (UA) and free fatty acid (FFA), etc., among which GDA-AB and IAA-Ab were determined by qualitative ELISA, FPG was determined by serum oxidase, GSP was determined by enzymatic method, HbA1c was determined by high performance liquid chromatography, and TC, TG, HDL-C, LDL-C, UA and FFA were determined by standard enzymes. All indexes were measured on Beckman AU5400 automatic analyzer according to standard laboratory methods.

### Statistical analysis

The statistical analyses of our data were performed by using SPSS 22.0 and R4.2 software. A two-tailed p-value less than 0.05 was considered as statistically significant. All continuous variables were presented as the mean ± standard deviation (`x ± s) or as median (lower quantile, upper quantile) [M (P25,P75)] and all categorical variables were expressed as a number (percentage constituent ratio,%). Two independent sample t test or nonparametric rank sum test (Wilcoxon) were used for comparisons of continuous variables and chi-square test (χ^2^) was used for comparisons of categorical variables between the two groups. In order to determine the independent predict factors of KPD, we first carried out univariate logistic regression analysis to preliminarily screen predict factors, and further conducted multivariate logistic regression analysis by adjusting for potential confounding factors to finally identify independent predict factors. Based on the independent predict factors of KPD, we used R4.2 software and rms package to construct a novel nomogram prediction model. In order to assess and validate the proposed model, we used three methods. First, receiver operation characteristic (ROC) curve was drawn, and the prediction performance was evaluated by calculating the area under curve (AUC) of ROC curve. Second, a corrected calibration curve containing 2000 bootstrap samples was used for the internal verification of the nomogram model to verify how well the predictive model was calibrated. Thirdly, the decision analysis curve (DCA) was generated to evaluate the clinical efficacy of the predictive model by analyzing the net benefit rate of KPD patients under different risk thresholds.

## Results

### Baseline clinical characteristics of participants

Compared with T2DM group, KPD group had a significantly lower value of ageand HDL-C and a significantly higher value of the proportion of male patients, family history of T2DM, HbA1c, FBG, FFA (all P < 0.05), there were no statistically significant differences in the proportion of overweight/obesity, serum UA, TC, TG, LDL-C, LP (a) and LAC between the two groups (all P > 0.05), as shown in **Table 1**.

**Table 1:** Comparison of clinical Characteristics between the KPD group and the T2DM group [n(%), x ± s, M (P25,P75)].

	KPD group (n=170)	T2DM group (n=228)	T//Z/X^2^	P value
Age (years)	44.13 ± 13.72	52.19 ± 12.97	5.983	<0.001
Gender (male/female)	137/33 (80.59%)	159/69 (69.74%)	6.017	0.014
Overweight/obesity (with/without)	91/79 (53.53%)	116/112 (50.88%)	0.274	0.600
Family history of T2DM (with/without)	86/84 (50.59%)	71/15 7(31.14%)	15.420	<0.001
HbA1c (%)	12.10 (10.48, 13.50)	9.10 (7.60, 10.90)	-10.358	<0.001
FBG (mmol/L)	10.70 (8.41, 13.39)	9.48 (7.73, 12.13)	-3.076	0.002
UA (umol/L)	338.70 (273.88, 421.73)	344.75 (284.35, 411.55)	-0.090	0.928
TC (mmol/L)	5.04 (4.33,6.00)	5.05 (4.35, 5.72)	-0.710	0.477
TG (mmol/L)	1.94 (1.23, 3.15)	1.95 (1.36, 2.76)	-0.172	0.864
HDL-C (mmol/L)	0.91 (0.75, 1.07)	1.01 (0.85, 1.17)	-3.849	<0.001
LDL-C (mmol/L)	3.07 ± 1.10	3.08 ± 0.92	0.132	0.895
LP (a) (mg/L)	69.45 (36.75, 179.38)	84.80 (40.80, 149.80)	-0.937	0.349
FFA (umol/L)	820.41 ± 220.58	490.75 ± 149.00	-17.776	<0.001
GSP (umol/L)	454.77 ± 120.38	449.33 ± 138.43	-6.614	<0.001
LAC (mmol/L)	3.07 (2.22, 3.77)	3.23 (2.50, 3.95)	-1.350	0.177

HbA1c, glycosylated hemoglobin A1C; FBG, fasting plasma glucose; UA, serum uric acid; TC, total cholesterol; TG, total triglycerides; HDL-C, high-density lipoprotein cholesterol; LDL-C, low-density lipoprotein cholesterol; LP (a), lipoprotein a; FFA, free fatty acid; GSP, glycosylated serum protein; LAC, Lactic acid.

### Single factor logistic regression analysis

Taking the occurrence of KPD (assignment: without KPD = 0, KPD = 1) as dependent variable, the predictive factors (assignment: female = 0, male = 1; without family history of T2DM = 0, with family history of T2DM = 1; other independent variables are introduced linearly) as independent variables, univariate logistic regression analysis was performed. The results revealed that age, proportion of male patients, family history of T2DM, HDL-C, HbA1c, FBG, FFA and GSP were predict factors for KPD (P<0.05).

### Multiple factor logistic regression analysis

Taking the occurrence of KPD as the dependent variable, the forward method was used to conduct multivariate logistic regression analysis on the eight significant variables in the above univariate regression analysis to screen the independent predict factors of KPD. The results exhibited that age, family history of T2DM, HbA1c, FFA became independent predict factors of KPD, as shown in **Table 2**.

**Table 2:** Multivariate logistic regression analysis of predict factors for KPD.

variable	β	SE	Wald	P	OR (95%CI)
Age	-0.041	0.013	10.845	0.001	0.959 (0.936, 0.983)
Family history of T2DM	0.961	0.343	7.844	0.005	2.614 (1.334, 5.122)
FFA	0.011	0.001	68.782	<0.001	1.011 (1.008, 1.014)
HbA1c	0.538	0.083	42.183	<0.001	1.712 (1.456, 2.014)

FFA, free fatty acid; HbA1c, glycosylated hemoglobin A1C.

### Construction of nomogram prediction model

**Figure 1:**
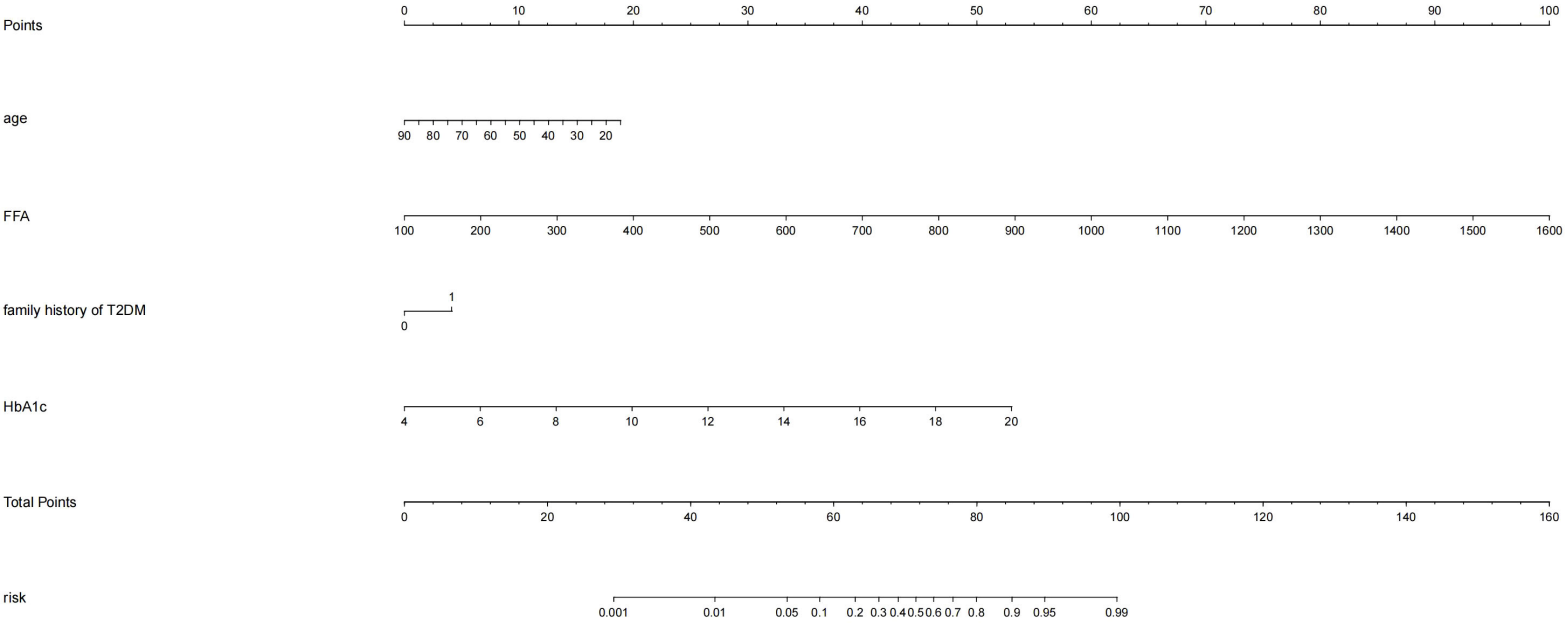
Development of the risk nomogram. The nomogram to predict the risk of Ketosis-prone type 2 diabetes (KPD) patients with T2DM was developed with the predictors including age, family history of T2DM, HbA1c, FFA Note: family history of T2DM:1;no family history of T2DM:0.

**Figure 2:**
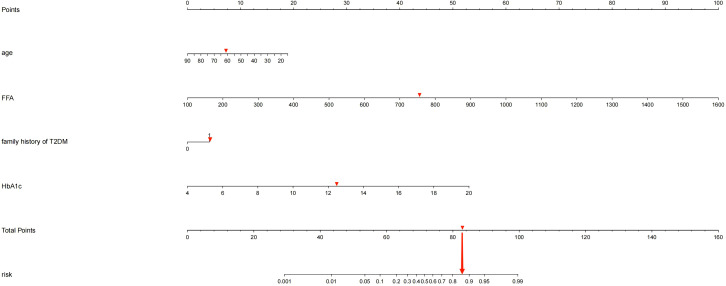
If a patient has a genetic family history of diabetes, was 62 years old, had an HbA1c of 12.3%, and had an FFA of 753.2umol/L, the probability of developing KPD is estimated to be 85.30%.

Based on the four independent predictors (age, family history of T2DM, HbA1c, FFA) according to the results of multivariate logistic regression analysis. The nomogram model for predicting the occurrence of KPD was established using the “lrm” function in the rms package of the R4.2 software to establish a Logistic regression model for the above four factors, and using the “plot” function to further develop a nomogram scoring system, the predictive factor with the highest score is FFA; followed by HbA1C; Age is ranked No.3 predictors, the last is the family history of type 2 diabetes (**Figure 1**). Among these variables, each factor was assigned a score on the point scale. After calculating the total score by adding the corresponding score value of each factor and positioning on the total score scale, a vertical line can be drawn down to obtain the predicted probability of KPD. The higher the score of total points, the greater the risk of KPD in patients with T2DM. As an example to better understand the predictive nomogram model, if the subject of T2DM is age of 62, HbA1c of 12.3%, FFA of 753.0 umol/L and has a family history of T2DM, the probability of KPD is estimated to be 85.3% (**Figure 2**).

### Validation and clinical use of nomogram prediction model

**Figure 3:**
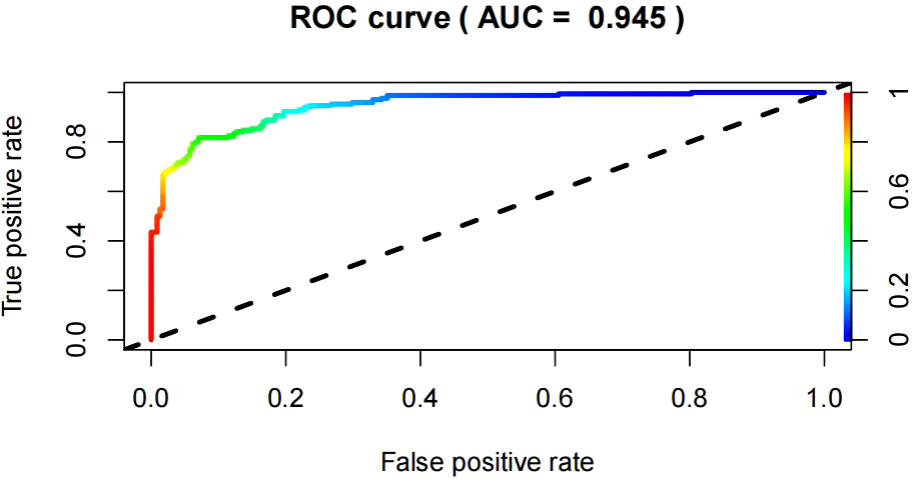
ROC curves of the Nomogram model predicting KPD occurrence.

**Figure 4:**
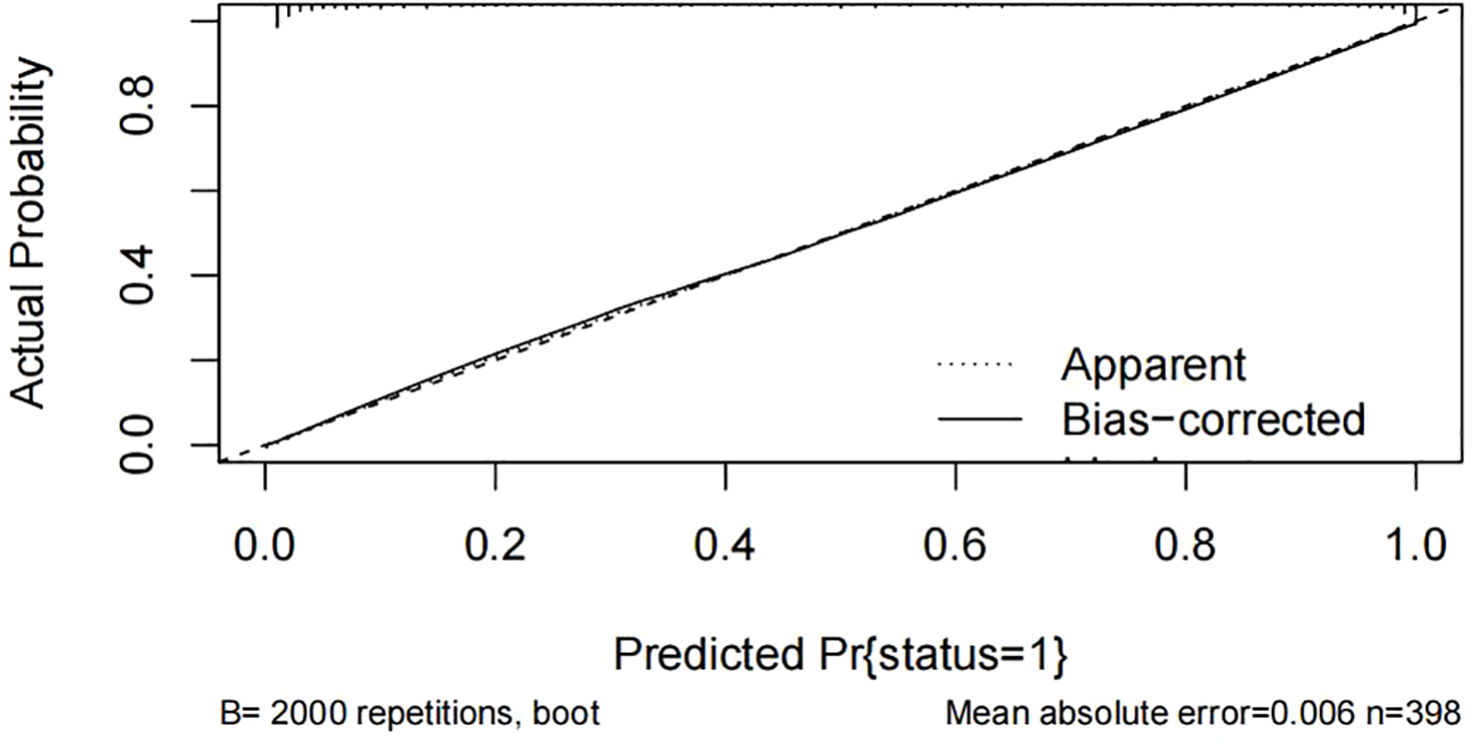
Correction curves in the Nomogram model.

**Figure 5:**
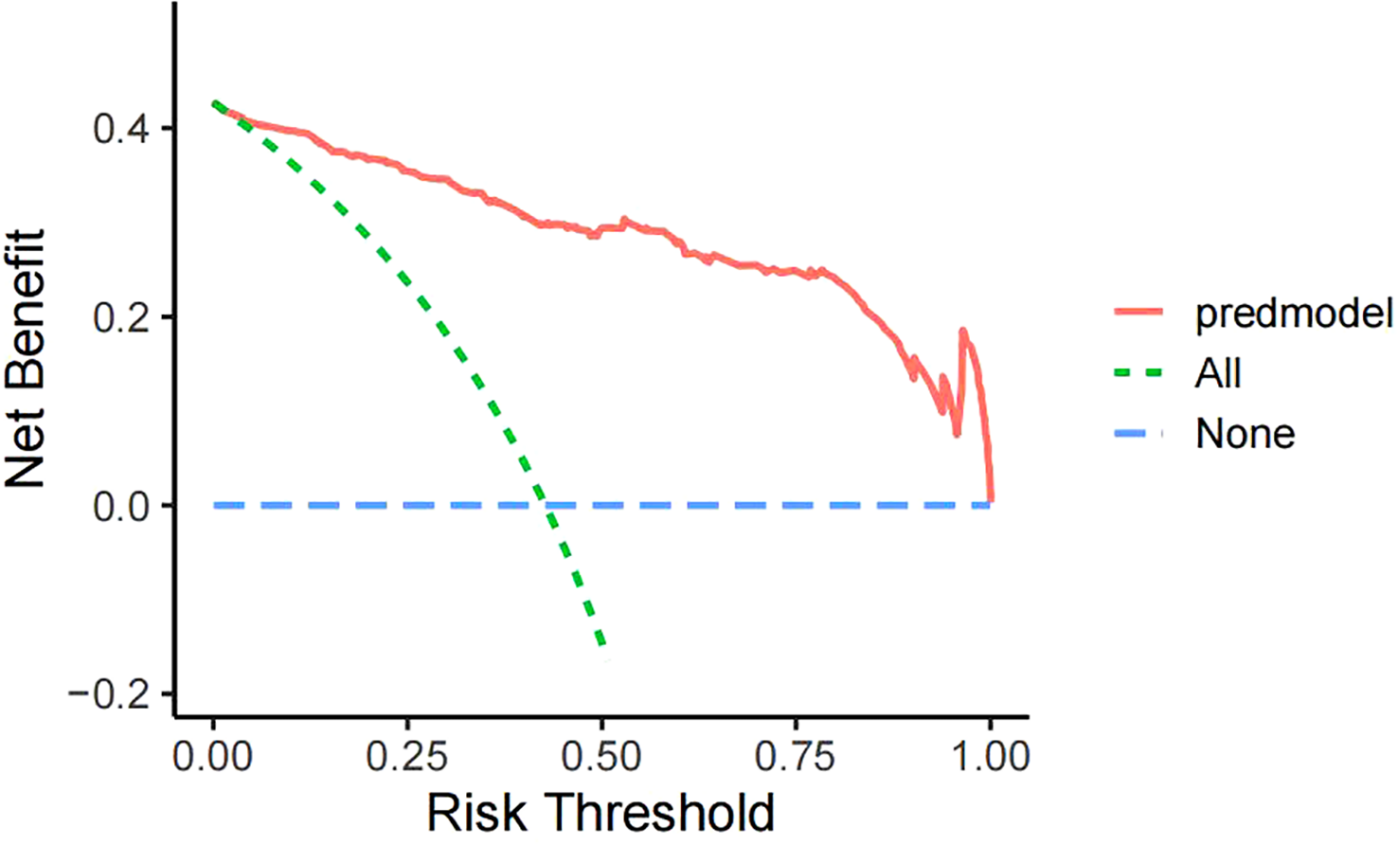
Clinical Decision Curve Analysis (DCA) in the Nomogram Model.

For the predictive model, the receiver operating characteristic (ROC) curve (**Figure 3**) was used to evaluate the performance of the model in predicting the risk of KPD and the area under curve (AUC) of ROC was 0.945, indicating that this model had a good prediction performance. The accuracy of the nomogram model was validated internally by bootstrap self-sampling method which was performed by generating 2,000 bootstrap samples to replace the original samples and repeating the calibration curve of the whole modeling process. The calibration curve of Nomogram (**Figure 4**) presented that there was a good agreement between the predicted probabilities and the actual probabilities with mean absolute error=0.006<0.05, and the accuracy and discrimination were both good. In addition, the DCA curve (**Figure 5**) showed that when the risk threshold was between 5% and 99%, the clinical net benefit obtained by patients is higher, indicating that the model had a wider range of application thresholds and was more clinically practical and accurate. Based on the the results from the above validation, the nomogram of KPD prediction model showed good prediction ability.

## Discussion

In recent years, with the increasing number of people with diabetes and its rising complication rate, an emerging special type of diabetes known as ketosis-prone type 2 diabetes (KPD) has become more and more common in clinical work (7). The peculiar manifestations of KPD are the presence of an acute severe hyperglycaemic state and marked hyperketaemia at the time of onset, but unlike classical type 1 diabetes, it lacks evidence of associated autoimmunity and has no apparent precipitating factors. The function of beta cells presents a transient impairment which can be recovered within a period of time after reasonable treatment. Insulin therapy can be discontinued and replaced with oral antidiabetic agents (14). However, due to its rapid onset and severe damage to β-cell function, acute hyperglycemia can trigger a vicious cycle leading to the development of acidosis, coma, and even death if left untreated (15). Given its harmful effects, there is an urgent need in clinical practice for an approach that enables early recognition and prevention of the occurrence of KPD, thereby reducing the incidence of associated adverse events and improving the long-term quality of life of patients. According to previous studies, many indicators of predict factors have been reported to be associated with the occurrence of KPD, such as age, gender, family history, BMI, HbA1c, C-peptide, TG, FFA and so on (16, 17). However, the vast majority of these studies only stay in the exploration of predict factors for KPD, and there is no systematic, easy, and efficient clinical tool for predicting the occurrence of KPD. Nomogram prediction model can synthesize multiple predict factors, demonstrate the different contribution of each predict factor to the occurrence of disease, and graphically predict the probability of outcome events, with good clinical practical performance, which is widely used in various fields (18). This study analyzed the predict factors for KPD occurrence on the basis of the inclusion of each clinical indicator and successfully constructed a nomogram model to predict the risk of KPD occurrence, and adopted a calibration curve with 2000 bootstrap samples for internal validation. The results showed an area under the curve of 0.945 for the ROC, indicating good discrimination, while the calibration curve showed a good degree of fit. Therefore, the model had good discrimination and consistency. The DCA curve indicates that the clinical net benefit obtained for patients is higher when the risk threshold is between 5% and 99%, indicating that the model has a broader range of thresholds for application and is more clinically practical and accurate.

Our results suggest that age, family history, HbA1c, FFA were independent predict factors for the development of KPD. HbA1c reflects glycemic control over the past 2-3 months and is a more stable indicator of long-term blood glucose control than fasting blood glucose and postprandial blood glucose (19). Studies have shown that HbA1c is significantly higher in patients with ketoacidosis, both in type 1 diabetes and T2DM, and in known and newly diagnosed diabetes (20). The average HbA1c level of KPD patients in this study was significantly higher than that of T2DM patients (12.1%& 9.1%), similar to previous studies (21), suggesting that KPD is likely to occur on a background of long-term chronic hyperglycemia. Severe and persistent glucotoxicity will have undesirable effects for insulin gene expression, proinsulin biosynthesis, and finally beta cell insulin secretion at each step that ultimately lead beta cells appeared dysfunctional (22). After the occurrence of hyperglycemia in patients with KPD, the function of islet beta cells declines sharply, thereby leading to the onset of ketosis. Patients with KPD cannot tolerate the transient diminution of islet function by hyperglycemia is also a major contributor to ketogenesis (23). T2DM patients maintain a certain amount of insulin secretion for a long period of time and can remain in a hyperglycemic state without ketosis (24). In patients with KPD, the function of islet beta cells is almost completely restored after normalizing their blood glucose by insulin treatment, so KPD is more sensitive to glucotoxicity (5). FFA was similarly shown to be independent predict factors for the development of KPD in the results of this study. KPD patients develop insulin resistance and hyperglycemia due to obesity and abnormal lipid metabolism, and the beta cells of these patients are prone to glucotoxicity (5, 7). The current study argues that the development of ketosis in KPD patients is largely attributed to a vicious cycle of glucolipotoxicity (25). A long hyperglycemic environment in the body and disturbed lipid metabolism caused by chronically elevated FFA reduced function and activity of beta cells, sharply decreases insulin secretion and aggravates persistent insulin resistance, leading to the onset of ketosis (26). In the case of severe insufficiency of insulin secretion in the circulation, the body is unable to properly utilize glucose and subsequently break down fat to produce energy for cellular utilization. After β-oxidation, a large amount of FFA generated from fat metabolism is condensed into ketones in liver tissue. Increased fatty acids can further impair islet function and increase obesity-related insulin resistance (27). Long-term chronic elevation of FFA can disturb the regulation of lipid metabolism, and lipid signals participate in FFA-induced apoptosis through receptors and intracellular mechanisms, thus leading to lower beta cell function and activity and higher blood sugar, thus forming a vicious cycle (28). It has been previously reported that KPD patients are usually obese middle-aged men with a strong family history of type 2 diabetes (7). In this study, 50.59% of KPD had a family history of diabetes, and the risk of developing KPD in patients with a family history was 2.614 times that of those without a family history, reflecting that KPD has an obvious genetic susceptibility. It may be associated with mutations in Ngn3 (Neurogenin3) and Pax4, which are closely related proendocrine genes in beta cell development (29). Alternatively, KPD may be associated with impaired defense mechanisms against oxidative stress due to mutations in the glucose-6-phosphate dehydrogenase (G6PD) gene, manifesting that β Cells are vulnerable to oxidative stress which can lead to acute insulin deficiency (30). Study found that young and middle-aged male patients with high blood ketone levels, are more likely to develop ketosis, lower than that of type 2 diabetes onset age (31), which are consistent with our results. The average age of KPD patients in this study was 44.13 ± 13.72 years old. The prevalence of obesity in young and middle-aged people is increased due to the accelerated pace of life, low physical activity, lack of regular healthy diet, and reduced exercise (32). On this basis, the sensitivity to glycolipid toxicity increased, the apoptosis and functional destruction of beta cells increased, and the ability of mitochondrial β-oxidation of fatty acids is reduced. When beyond their upper capacity limits, accelerated ketogenesis in the liver greater than tissue utilization may be responsible for ketogenesi (28).

As far as we know, this Nomogram model can be used to visually predict the risk of KPD occurrence with high accuracy based on various independent predict factors. However, the limitation of this study is that the obtained sample was derived from a single center and the sample size was limited, although the nomogram model exhibited better accuracy, which still needs to be further prospective multicenter and external validation to improve the reliability of the model and increase the clinical usefulness. In addition, this study was of a retrospective design, not all clinical observation variables were included due to the limited availability of data and the presence of other potential predict factors, and further refinement is needed to explore a more comprehensive and accurate intensive model.

## Conclusion

In summary, after evaluating multiple variables and screening independent predict factors through multivariate logistic regression analysis, we demonstrated that age, family history of T2DM, HbA1c and FFA were the independent predict factors of KPD in patients with newly diagnosed T2DM. Combining with these four feasible clinical variables, a novel nomogram for the risk of KPD among patients with T2DM were constructed and internal validation was carried out. The developed nomogram exhibited a great accurate value and behaved good discrimination as a remarkable intuitive risk assessment and prediction tool for KPD prediction. As one of the acute complications of diabetes, ketosis will produce serious adverse consequences if it is not detected in time and treated actively. In view of its serious harm and possible adverse events, rapid diagnosis and early and timely glucose lowering therapy are clinically necessary for diabetes related acute metabolic complications. According to the above evaluation results, this simple and intuitive nomogram improves the early prediction and identification of high-risk groups, and help clinicians and T2DM patients to estimate the risk of KPD, so as to actively adopt targeted treatments on medical interventions to prevent the emergence of acute complications in time.

## Data availability statement

The original contributions presented in the study are included in the article/**Supplementary Material**. Further inquiries can be directed to the corresponding author.

## Ethics statement

The studies involving humans were approved by Ethics Committee of Wuhan Fourth Hospital. The studies were conducted in accordance with the local legislation and institutional requirements. Written informed consent for participation was not required from the participants or the participants’ legal guardians/next of kin in accordance with the national legislation and institutional requirements.

## Author contributions

RM and BP contributed equally to this manuscript, and BP is the co-first author of this article. RM conceived and designed the study, analyzed the data, and drafted the first manuscript. BP, YL were involved in the collection of the data and preparation of figures and tables. BP critically revised the manuscript. All authors contributed to the article and approved the submitted version.
